# Nematic and Smectic Phases with Proper Ferroelectric Order

**DOI:** 10.1002/advs.202409754

**Published:** 2024-11-25

**Authors:** Grant J. Strachan, Ewa Górecka, Jadwiga Szydłowska, Anna Makal, Damian Pociecha

**Affiliations:** ^1^ Faculty of Chemistry University of Warsaw Zwirki i Wigury 101 Warsaw 02–089 Poland

**Keywords:** dielectric constant, ferroelectric, liquid crystals

## Abstract

A material showing a sequence of three ferroelectric liquid crystalline phases below the paraelectric nematic phase is synthesized and studied. The polar order of molecules appearing due to the dipole–dipole interactions in the ferroelectric nematic, N_F_, phase is preserved also in the smectic phases: orthogonal SmA_F_ and tilted SmC_F_. The ferroelectric ground state of both smectic phases is confirmed by their second harmonic generation (SHG) activity and polarization switching. In the SmC_F_ phase the polarization becomes oriented to the electric field by decreasing the tilt angle to zero. Although both smectic phases are ferroelectric in nature, their dielectric response is found to be very different.

## Graphical abstract

The evolution of polar and lamellar order is seen in the phase sequence of a new ferroelectric liquid crystal, without intermediate antiferroelectric or paraelectric behavior, broadening the ferroelectric realm of liquid crystal phases.

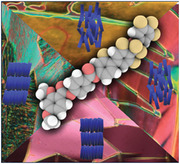

## 1 Introduction

Ferroelectricity is a material property that refers to the presence of spontaneous electric polarization which is reversible on the application of an electric field. It was first discovered in Rochelle salt by J. Valasek in 1921,^[^ ^1^ ^]^ and today, there are only ≈ 300 known ferroelectric crystals, making it still a relatively uncommon property for crystals. Ferroelectricity is also relatively rare in soft matter. Ferroelectric polymers maintain a permanent electric polarization due to the all‐*trans* conformation of polymer chains and thus parallel ordering of transverse dipole moments, and the most studied example is polyvinylidene fluoride.^[^ ^2^ ^]^ In the 1970s, ferroelectric properties were also discovered in tilted smectic phases composed of chiral molecules.^[^ ^3^ ^]^ In subsequent years, antiferroelectric chiral SmC phases^[^ ^4^ ^]^ and polar properties of achiral bent‐core mesogens^[^ ^5^ ^]^ were also discovered. However, all these liquid crystals are examples of improper ferroelectrics, in which the polar order is a secondary effect. The ordering of dipole moments is induced by steric interactions between the molecules and therefore is usually weak.

**Figure 1:**
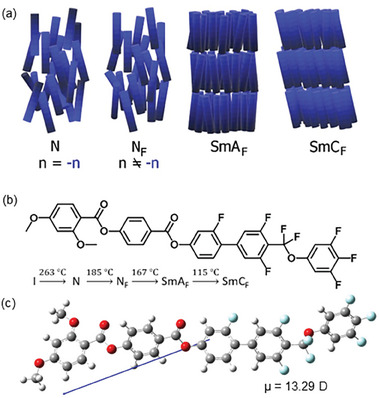
a) Schematic drawing showing the arrangement of polar molecules in N, N_F_, SmA_F,_ and SmC_F_ phases. b) The molecular structure of the mesogen reported here, with the phase transition temperatures. c) The minimum energy conformation calculated at the B3LYP‐GD3BJ/cc‐pVTZ level of DFT with an arrow showing the direction of the molecular dipole moment.

For many years, it was believed that dipole–dipole interactions themselves were too weak to produce long‐range polar ordering in the liquid state, and the polar order would be disrupted by thermal fluctuations. This common belief was overturned recently by the discovery of the ferroelectric nematic (N_F_) phase,^[^ ^6^, ^7^, ^8^ ^]^ in which the spontaneous electric polarization vector is along the director. In the N_F_ phase the polar ordering is exceptionally strong, while the viscosity is not much different from that of regular liquids, making these materials interesting for future applications. At first glance, it seems that longitudinal polar order should be even easier to obtain in the smectic phase than in the nematic phase, as thermal fluctuations in lamellar systems are strongly suppressed. The ferroelectric orthogonal smectic (SmA_F_) phase was first claimed to have been discovered in 1991,^[^ ^9^ ^]^ but this finding turned out to be premature.^[^ ^10^ ^]^ Since the discovery of the N_F_ phase, attention has returned to the search for different ferroelectric smectic phases in combination with the polar N_F_ phase. This has proved challenging because the requirements for polar order and smectic order are contradictory. Smectic order generally requires molecules to have long tails to enhance the self‐segregation of mesogenic cores and alkyl chains that provide the main mechanism of layer formation. However, this inevitably increases the distance between interacting dipoles, which weakens their tendency for order. Therefore, only a limited number of mesogens that show a sequence of polar nematic and smectic phases are known so far.^[^ ^11^, ^12^, ^13^, ^14^, ^15^, ^16^, ^17^, ^18^ ^]^ In this work, we report the phase behavior and ferroelectric properties of a new liquid crystalline material, which shows the sequence of three polar mesophases: N_F_, SmA_F_, and SmC_F_ below the paraelectric N phase (**Figure** **1**a). This allows us, for the first time, to follow the development of polar order and its coupling to positional order and tilt.

The molecular structure of the material studied here is given in Figure 1 together with its calculated dipole moment, phase sequence, and phase transition temperatures determined from calorimetric studies (Figure S7, Supporting Information). It shares common fragments with archetypical ferronematogens, RM734,^[^ ^6^ ^]^ DIO,^[^ ^7^ ^]^ and UUQU‐4N.^[^ ^19^ ^]^

## 2 Results

### 2.1 Identification of Liquid Crystalline Phases

**Figure 2:**
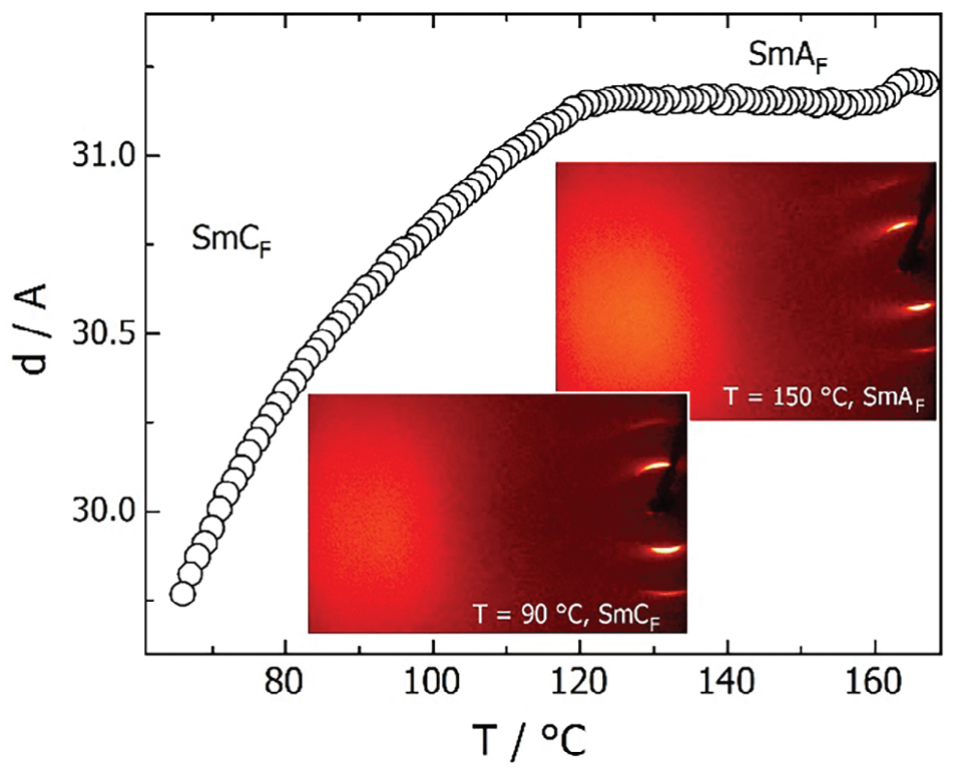
Layer spacing versus temperature; in the inset the 2D XRD patterns registered in SmA_F_ and SmC_F_ phases, confirming liquid‐like in‐plane order of molecules in both phases.

The material shows a sequence of four liquid crystalline phases. The two higher temperature phases were confirmed to be nematic as only short‐range positional order of molecules is observed by X‐ray diffraction (XRD). In the two lower temperature phases the low‐angle XRD signal narrows to become limited only by instrumental broadening, reflecting the formation of a long‐range lamellar structure and indicating that these are both smectic phases (**Figure** **2**). In the upper temperature smectic phase, the position of the signal is nearly constant and corresponds to a layer thickness that closely matches the molecular length (30 Å) determined in the crystalline state by single crystal XRD (see SI). This is typical for a smectic A phase. In the lower temperature phase, the layer spacing gradually decreases on lowering the temperature, indicating the tilting of molecules within the layers and suggesting that this is a SmC phase. The tilt angle estimated from the change in layer thickness reaches ≈15 degrees 30 K below the SmA – SmC phase transition. It should also be noted that in all LC phases the high‐angle diffraction signal was diffuse, showing no long‐range positional correlations along the short molecular axes, consistent with a sequence of nematic and liquid‐like smectic phases.

**Figure 3:**
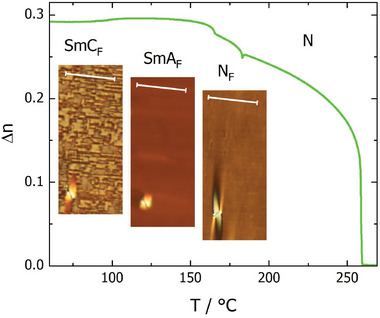
Optical birefringence, Δn, versus temperature. The dip at the N‐N_F_ transition reflects the apparent decrease of the orientational order parameter, S, by ≈0.01. The step‐like increase of Δn at the N_F_‐SmA_F_ phase transition corresponds to S changing from 0.78 to 0.81. Inset: the optical textures of N_F_, SmA_F,_ and SmC_F_ phases taken in a 1.8‐µm‐thick cell with planar anchoring and parallel rubbing direction on both surfaces. Scale bars correspond to 50 µm and show the rubbing direction, which is slightly rotated from the polarizer direction.

**Figure 4:**
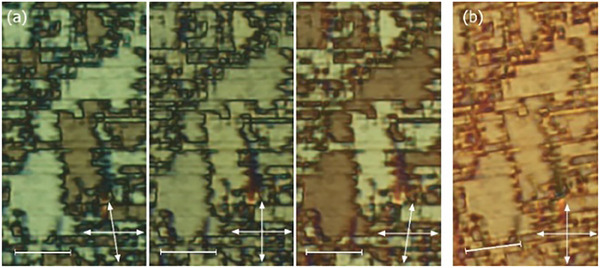
The optical texture of the SmC_F_ phase observed with a polarized light microscope in a 1.6‐µm‐thick cell with planar anchoring. Scale bars correspond to 20 µm and are placed along the rubbing direction. a) Slight de‐crossing of polarizers (arrows) distinguishes domains with opposite optical activity. b) Upon rotating the sample with respect to crossed polarizers all domains change brightness in the same manner. This indicates that the direction of the optical axis is the same in the different domains and thus the domains are not defined by different tilt directions.

**Figure 5:**
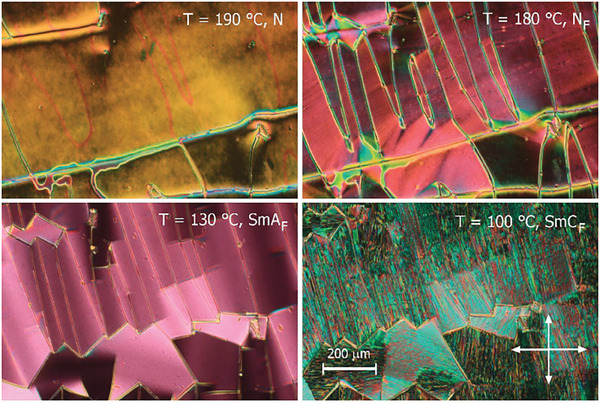
Optical textures observed with polarized light microscopy in LC phases of the reported compound in a 5‐µm‐thick cell with homeotropic anchoring. Arrows indicate the polarizer's direction.

The sequence of LC phases was also followed by observations of characteristic optical textures with polarized‐light optical microscopy and measurements of the optical birefringence. In thin (1.5‐3 µm) cells with planar anchoring and parallel rubbing on both surfaces both nematic phases and the smectic A phase gave a uniform texture, with the optical axis along the rubbing direction (**Figure** **3**). The textures seen for the higher temperature nematic phase are consistent with its identification as a conventional nematic, N, phase. On cooling into the lower temperature nematic phase several conical defects were formed, anchored at the glass pillars that are cell spacers, and such defects are characteristic of the ferroelectric nematic phase. At the transition from the orthogonal to the tilted smectic phase the uniform texture breaks into small domains, in which the optical axis departs from the rubbing direction. These domains have a characteristic blocky shape with longer sides along the rubbing direction, and in some areas, they also show weak optical activity (**Figure** **4**). In optically active domains the molecular orientation at the lower and upper surfaces of the cell differs and inside the cell molecules twist to connect the surface layers. Interestingly, all the liquid crystalline phases showed birefringent textures in cells with homeotropic anchoring (**Figure** **5**). The schlieren texture observed in the nematic phase transformed into mosaic‐like textures in the lower temperature phases, composed of clearly separated domains. The domains were smooth in the N_F_ and SmA_F_ phases and broke into numerous stripes in the SmC_F_ phase. It should be noted that neither typical focal conic nor fan‐shaped textures were observed in the smectic phases, suggesting that polar order is preserved on cooling from the N_F_ phase.^[^ ^14^ ^]^

The optical birefringence, Δn, increases continuously in the nematic phase, following a critical, power‐law dependence of the orientational order of the molecules (Figure 3). The trend continues also in the N_F_ phase, however close to the N‐N_F_ transition a pronounced dip in Δn is observed. It appears that the development of polar order is accompanied by strong orientational order fluctuations (splay deformations), lowering the effective optical anisotropy.^[^ ^20^ ^]^ The N_F_‐SmA_F_ phase transition is marked by a step‐like increase of birefringence, indicating that the formation of long‐range positional order is accompanied by a small increase in the orientational order of molecules; the order parameter S jumps from 0.78 to 0.81, and such a discontinuity is often observed at weakly first‐order transitions. At the SmA_F_‐SmC_F_ phase transition, the measured Δn values decrease, however this apparent behavior may be ascribed to distortion of the uniform texture as described above.

Calorimetric studies revealed that three of the observed LC phases are enantiotropic, the pristine crystal melts at 145 °C into the SmA_F_ phase, and the monotropic SmC_F_ phase can be observed due to the supercooling effect (Figure S7, Supporting Information). The N_F_‐N phase transition was accompanied by a jump in the heat capacity, characteristic of a second‐order phase transition, while at the SmA_F_ – N_F_ phase transition a small enthalpy peak was registered, which is characteristic for a weakly first‐order transition. These findings are consistent with the optical studies. Despite the clearly visible optical texture changes at the SmA_F_‐SmC_F_ phase transition, no change in the heat capacity was detected in calorimetric measurements, suggesting that the phase transition is second order with a change in heat capacity below the detection limit of the DSC.

From these observations, we can see that the studied material shows a sequence of liquid crystal phases with gradually increasing molecular order: nematic → orthogonal smectic → tilted smectic. Taking into account the strong molecular dipole moment, the polar properties of the LC phases should also be considered. While the ferroelectric nature of the lower temperature nematic phase was indicated by the observed optical textures characteristic of the N_F_ phase, the properties of the smectic phases require further investigation, and this will be discussed in the following section.

### 2.2 Polar Nature of the Liquid Crystal Phases

The studied molecule is highly polar, and its dipole moment was calculated to be 13.29 D (using DFT at the B3LYP‐GD3BJ/cc‐pVTZ level). While it must be remembered that this calculation considers only a single conformation of an isolated molecule in the gas phase, and hence may be a slight overestimation of the true value of the dipole moment for an average conformation in the LC phases, the calculated value is consistent with those reported for similar ferroelectric LCs, and much greater than those of conventional mesogenic materials, e.g. ca. 4 D for 5CB.^[^ ^21^ ^]^ The dipole moment in the studied molecule is offset only slightly from the long molecular axis (Figure 1), as reported for similar molecules.^[^ ^11^, ^16^ ^]^ Therefore, in polar phases, in which molecules rotate freely around a long axis the spontaneous electric polarization vector is expected to lie along the director.

**Figure 6:**
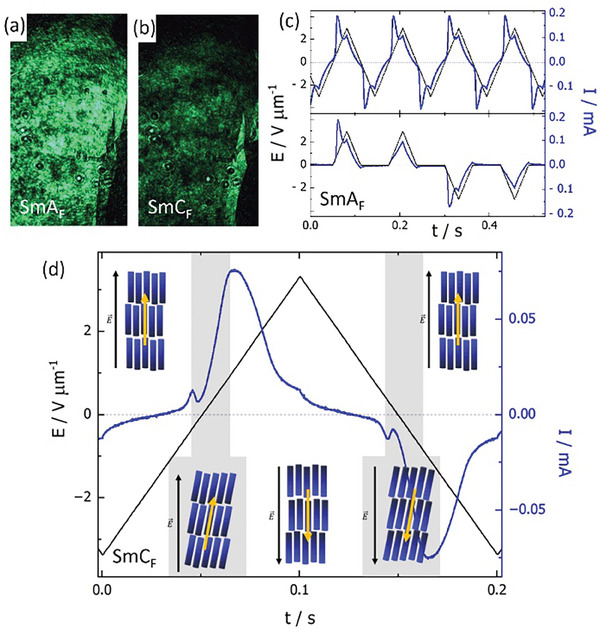
Images taken with SHG microscope in ground state (no applied voltage) of a) SmA_F_ and b) SmC_F_ phases for a sample prepared in a cell with planar anchoring. Incident IR (λ = 1064 nm) radiation resulted in emission of green (SH) light in both phases, proving their ferroelectric properties. c) Switching current in the SmA_F_ phase (blue lines) under the application of triangular and modified triangular wave voltage (black lines). Application of two successive pulses of the same sign results in the current peak appearing only in the first pulse, confirming the stable ferroelectric ground state of the phase. d) The switching current in the SmC_F_ phase (blue line) under application of triangular wave voltage (black line), showing the main switching peak and the additional smaller peak at low voltages. A schematic representation of the proposed two‐step switching process in the SmC_F_ phase is given, with the black arrow representing the direction of the applied electric field and the yellow arrow corresponding to the direction of the spontaneous polarization.

It should be noted that all the LC phases below the N phase are SHG active at zero electric field (**Figure** **6**a,b), which confirms the ferroelectric character of their ground state (SmA_F_ and SmC_F_). Their response to an applied electric field has been studied by observation of optical texture changes in cells with transparent electrodes. The application of an electric field across the cell thickness in the N_F_ and SmA_F_ phases induces a homeotropic texture as the polarization, and thus the director, aligns along the electric field (Figure S8, Supporting Information). In the N_F_ phase, when the field is reduced to zero the sample relaxes immediately to a birefringent state with polarization inclined to the cell surface to reduce surface‐bound charges.^[^ ^18^ ^]^ In the SmA_F_ phase the relaxation to a birefringent texture takes place but the process is slow (taking several seconds) as the reorientation of polarization requires layer rotation. In the SmC_F_ phase, applying an electric field also leads to a non‐birefringent state, however, this occurs through an intermediate state with reduced birefringence (Figure S8, Supporting Information). This suggests a two‐step switching mechanism, involving the reversal of polarization through reorientation of the layers, and in addition, a second process related to the removal or restoration of the director tilt.

The ferroelectric nature of the smectic phases has been further confirmed by observation of a clear switching current peak when AC voltage is applied across the sample (Figure S6 and Figure S9, Supporting Information). The spontaneous electric polarization, calculated from the peak area, increases in the N_F_ phase and reaches ≈5 µC cm^−2^ in the smectic phases. Moreover, the ferroelectric ground state of the smectic A_F_ phase was confirmed by studying the switching behavior using a modified triangular wave voltage (Figure 6c). In this experiment two successive positive voltage pulses (separated by a period with zero voltage and followed by two negative ones) were applied and the switching current peak was observed only for the first pulse of each sequence. This indicates that reducing the voltage from the maximum value to zero preserves the ferroelectric arrangement of dipoles.

In the SmC_F_ phase, under application of triangular‐wave voltage, apart from the main switching current peak due to the reversal of polarization, an additional small peak is detected, positioned close to 0 V (Figure 6d). Such a peak has been observed in other recently reported polar SmC phases.^[^ ^12^, ^22^ ^]^ This small peak decreases on heating and finally disappears on entering the SmA_F_ phase (Figure S9, Supporting Information). The underlying mechanism producing this signal must be a very low‐energy process. One possibility may be that this corresponds to the reappearance of the molecular tilt within the SmC_F_ phase, and a sketch of such a possibility is given in Figure 6d. The whole switching sequence in the SmC_F_ phase would involve three steps: first, the polarization within the layers aligns with the electric field when a high voltage is applied – in this state the tilt angle is removed and the phase becomes orthogonal; second, when the electric field is reduced, molecular tilt is regained, which changes the electric polarization along the layer normal and causes the small current peak; and finally, after reversal and increase of the applied voltage the polarization switches and realigns with the applied electric field, giving rise to the main recorded current peak.

**Figure 7:**
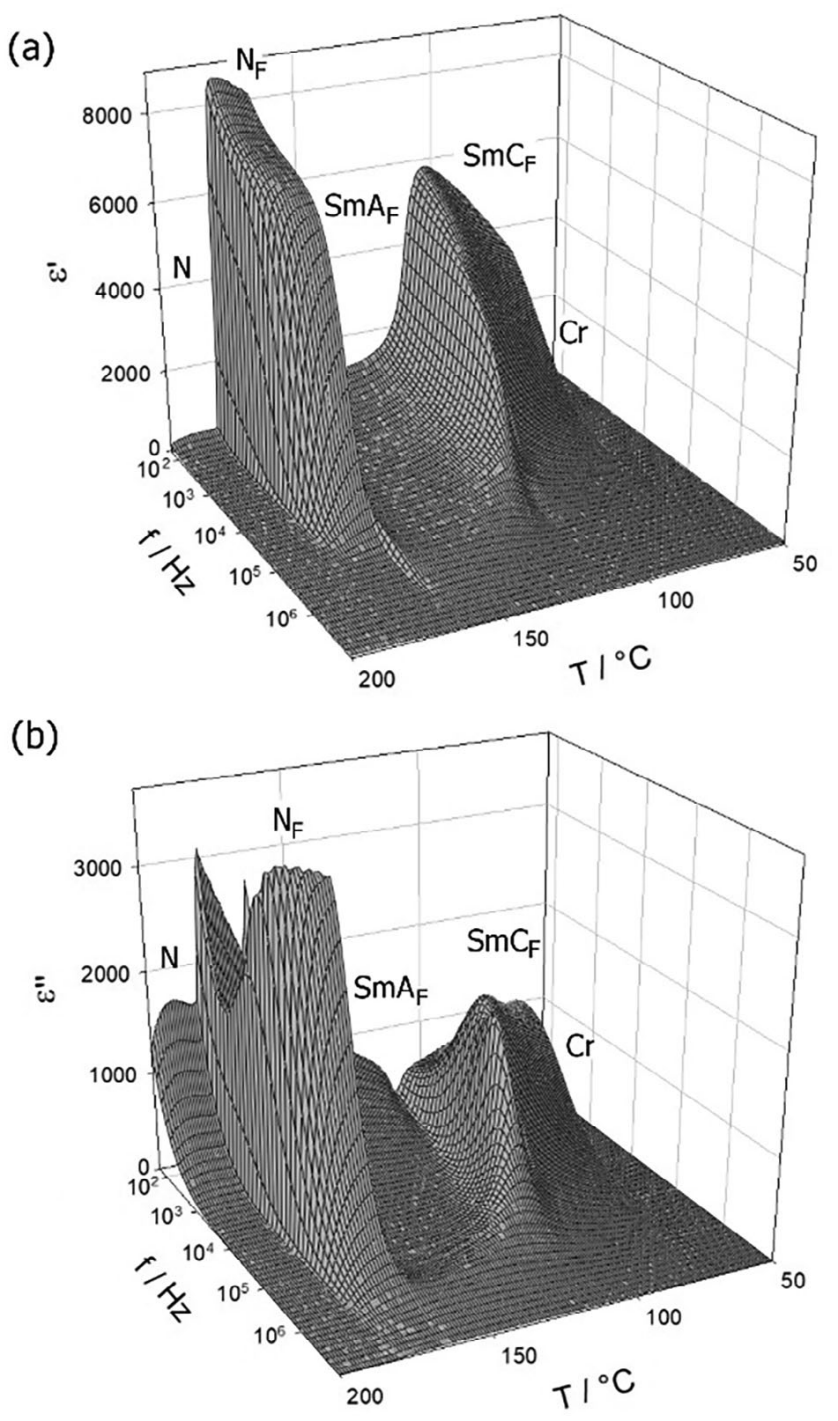
Real a) and imaginary b) parts of apparent dielectric permittivity measured in a 10‐µm‐thick cell with gold electrodes.

Finally, dielectric spectroscopy studies were conducted to follow the characteristic fluctuations of polar order in the LC phases. Although the interpretation of dielectric measurements for strongly polar phases is not straightforward,^[^ ^23^, ^24^ ^]^ and measured values of both the position and dielectric strength of relaxation modes are influenced by the thickness and type of the cells used, the relative changes in dielectric constant largely reflect the material properties. The studied compound was examined in a 10‐µm‐thick cell with gold electrodes and no polymer aligning layers, to avoid contribution from the polymer layer capacitance. In such cells the orientation of the director with respect to the measuring electric field is random. In the N_F_ phase, a strong, nearly temperature‐independent dielectric response was detected, with a relaxation frequency of ≈10^4^ Hz (**Figure** **7**). In the SmA_F_ phase, a much lower permittivity is measured in the whole tested frequency range, 10–10^7^ Hz. On lowering the temperature and upon approaching the tilted smectic phase, a weak, high‐frequency mode starts to build in the SmA_F_ phase, with a relaxation frequency that critically decreases, and a mode strength that critically increases; such behavior can be ascribed to the softening of the tilt angle fluctuations. In the SmC_F_ phase the soft mode condenses and a strong dielectric response is restored, with relaxation at ≈10^4^ Hz. The low permittivity in the ferroelectric SmA_F_ phase in comparison to the strong response in the SmC_F_ phase might be explained by the different types of polar fluctuations possible in these phases. In the non‐tilted smectic A_F_ phase, two basic fluctuation modes can be considered: changes in the magnitude of the polarization vector and undulation of the polar layers. The first mechanism is active only when the system is in the close vicinity of a transition to a paraelectric phase, which is not the case in the material studied here. The second mechanism involves layer undulations, which should be strongly suppressed in highly polar systems as they produce bound charges related to the local splay of the polarization vector. In the SmC_F_ phase, an additional fluctuation mechanism is activated due to molecular tilt – the collective rotation of molecules on the tilt cone. Due to the lower energy required for such fluctuations, the strong dielectric response is restored in this phase.

Combining the insights from the measurements described above and the properties of the three polar phases studied, we can consider the relation between polar and orientational order in these phases. In all three phases, the dipole moment is expected to follow, approximately, the director of the LC phase. As such, in the orthogonal smectic A_F_ phase the polarization is along the layer normal, while it is inclined by a tilt angle in the SmC_F_ phase. Such an arrangement allows the development of lamellar order without the need for any dramatic reorganization of the polar structure between the different LC phases, allowing for the observed sequence of ferroelectric phases without intermediate antiferroelectric or paraelectric phases.

## 3 Conclusion

In summary, the material studied here shows a sequence of three ferroelectric phases: N_F_‐SmA_F_‐SmC_F_, and our results show that the development of lamellar structure has only a minor effect on the polar order. The polar, ferroelectric character is uninterrupted across the SmC_F_, SmA_F,_ and N_F_ phases. While the N_F_‐SmA_F_ phase transition is weakly first order, the SmA_F_‐SmC_F_ transition is second order. Although the polarization value determined from the switching current is similar in both ferroelectric smectic phases, their dielectric response is very different. In the orthogonal ferroelectric smectic phase polar fluctuations are strongly suppressed, while in the tilted SmC_F_ phase collective rotation of molecules on a tilt cone gives rise to a strong dielectric response. Both smectic phases easily respond to an electric field; in the SmA_F_ phase under a reversing electric field switching takes place between the two optically homeotropic states, while in the SmC_F_ phase an intermediate state with schlieren texture is formed with gradually reduced birefringence, showing that under an electric field the cone angle is reduced to zero.

## 4 Experimental Section

The full synthetic and chemical characterization details are described in the Supporting Information, as well as molecular structure and crystal structure parameters obtained from single crystal X‐ray diffraction (XRD) experiment (Figures S5–S7, Supporting Information).

[CCDC 2375621 contains the supporting crystallographic data for this paper. These data can be obtained free of charge from The Cambridge Crystallographic Data Centre via www.ccdc.cam.ac.uk/data_request/cif.]

## Conflict of Interest

The authors declare no conflict of interest.

## Supporting information



Supporting Information

## Data Availability

The data that support the findings of this study are available in the supplementary material of this article.
