# Supplementary material for: Nematic and Smectic Phases with Proper Ferroelectric Order
Source: Adv Sci (Weinh). 2024 Nov 25;12(3):2409754. doi: 10.1002/advs.202409754 (PMC11744557; doi:10.1002/advs.202409754)
Supplement: Supplementary file 1 — Supporting Information [file ADVS-12-2409754-s001.pdf]

ADVANCED SCIENCE
Open Access

Supporting Information

for *Adv. Sci.*, DOI 10.1002/advs.202409754

Nematic and Smectic Phases with Proper Ferroelectric Order

Grant J. Strachan, Ewa Górecka, Jadwiga Szydłowska, Anna Makal and Damian Pociecha*

Nematic and Smectic Phases with Proper Ferroelectric Order

Grant J. Strachan, Ewa Górecka, Jadwiga Szydłowska, Anna Makal, Damian Pociecha

Experimental methods 1
Synthetic procedures and chemical characterisation 2
Single crystal X-ray diffraction........ 7
Supplementary figures 13
Supplemental references 14

Experimental Methods

Transition temperatures and the associated enthalpy changes were measured by differential scanning calorimetry using a TA DSC Q200 instrument. Measurements were performed under a nitrogen atmosphere with a heating/cooling rate of 10 K min^{-1}, unless otherwise specified.

Observations of optical textures of liquid crystalline phases was carried out by polarised-light optical microscopy using a Zeiss AxioImager.A2m microscope equipped with a Linkam heating stage.

Optical birefringence was measured with a setup based on a photoelastic modulator (PEM-90, Hinds) working at a modulation frequency f = 50 kHz; as a light source a halogen lamp (Hamamatsu LC8) equipped with narrow bandpass filters was used. The transmitted light intensity was monitored with a photodiode (FLC Electronics PIN-20) and the signal was deconvoluted with a lock-in amplifier (EG&G 7265) into 1f and 2f components to yield a retardation induced by the sample. Knowing the sample thickness, the retardation was recalculated into optical birefringence. Samples were prepared in 1.6-μm-thick cells with planar anchoring. The alignment quality was checked prior to measurement by inspection under the polarised-light optical microscope.

X-ray diffraction measurements of samples in liquid crystalline phases were carried out using a Bruker D8 GADDS system, equipped with micro-focus-type X-ray source with Cu anode and dedicated optics and VANTEC2000 area detector. Small angle diffraction experiments were performed on a Bruker Nanostar system (IμS microfocus source with copper target, MRI heating stage, Vantec 2000 area detector).

Single-crystal X-ray diffraction data were collected on a SuperNova diffractometer with micro-focus sealed source of MoKα X-ray radiation (λ = 0.71073 Å) and CCD Eos detector. Single crystals of studied compound were obtained from a chloroform solution using hexane as an antisolvent. A suitable crystal – a colorless prism - was mounted on a nylon loop with a trace of ParatoneN oil. The crystal was kept at 120.00(10) K during data collection in cold nitrogen stream using Oxford Cryosystems device. Data reduction was performed with CrysAlisPro.[1] Gaussian absorption correction was applied using spherical harmonics with SCALE3 ABSPACK algorithm. Using Olex2,[2] the structure was solved with the olex2.solve[3] program using Charge Flipping and refined with the olex2.refine[3] package using Gauss-Newton

minimization. H-atom positions were identifiable from a difference Fourier map but were refined with distances restrained to standardized values and the atomic displacement parameters (ADP-s) of H atoms were restrained as 'riding' on the displacement parameters of the covalently bound non-H atoms. A static disorder concerning the position of the fluorine F8 was refined, yielding 93% of the major component (F8 bound to C19) and 7% of the minor component with F8a bound to C15. Similarity restraints were applied for C – F distances and F displacement parameters of both disorder components.

Spontaneous electric polarisation was determined by integration of the current peaks recorded during polarization switching upon applying a triangular-wave voltage. 3- to 10-µm-thick cells with ITO or gold electrodes and no polymer aligning layers were used, and the switching current was determined by recording the voltage drop on a resistor connected in series with the sample.

The SHG response was investigated using a microscopic setup based on a solid-state laser EKSPLA NL202. Laser pulses (9 ns) at a 10 Hz repetition rate and max. 2 mJ pulse energy at λ=1064 nm were applied. The pulse energy was adjusted for each sample to avoid its decomposition. The infra-red beam was incident onto a LC homogenous cell of thickness 5 µm. An IR pass filter was placed at the entrance to the sample and a green pass filter at the exit of the sample.

The complex dielectric permittivity, ε*, was measured using a Solartron 1260 impedance analyser, in the 1 Hz −10 MHz frequency range, and a probe voltage of 50 mV. The material was placed in a 5- or 10-µm-thick glass cell with gold electrodes. Cells without polymer aligning layers were used, as the presence of the thin (~10 nm) polyimide layers at the cell surfaces acts as an additional high capacitance capacitor in a series circuit with the capacitor filled with the LC sample, which for materials with very high values of permittivity, may strongly affect the measured permittivity of the LC phases. Lack of a surfactant layer resulted in a random configuration of the director in the LC phases.

DFT geometry optimization was carried out at the B3LYP- GD3BJ/cc-pVTZ level of theory using Gaussian 16 (Revision C.01)[4] on the Ares cluster of the Polish high-performance computing infrastructure PLGrid (HPC Center: ACK Cyfronet AGH. Following geometry optimization, a frequency calculation was used to confirm that the obtained structure was at an energy minimum.

Synthetic Procedures and Structural Characterisation

Unless otherwise stated, all materials were obtained from commercial sources and used without further purification.

Reactions were monitored using thin layer chromatography (TLC) using aluminium-backed plates with a coating of Merck Kieselgel 60 F254 silica and an appropriate solvent system. Spots were visualised using UV light (254 nm). Flash column chromatography was carried out using silica grade 60 Å 40-63 micron.

FT-IR spectra were obtained using a Nicolet iS50 FT-IR spectrometer. ^{1}H, ^{19}F, and ^{13}C NMR spectra were recorded on a 400 MHz Agilent NMR spectrometer using either $CDCl_3$ or DMSO-*d*$_6$ as solvent and using residual non-deuterated trace solvents as reference. Chemical shifts (δ) are given in ppm relative to TMS (δ = 0.00 ppm). Mass spectroscopy was conducted on a Micromass LCT instrument.

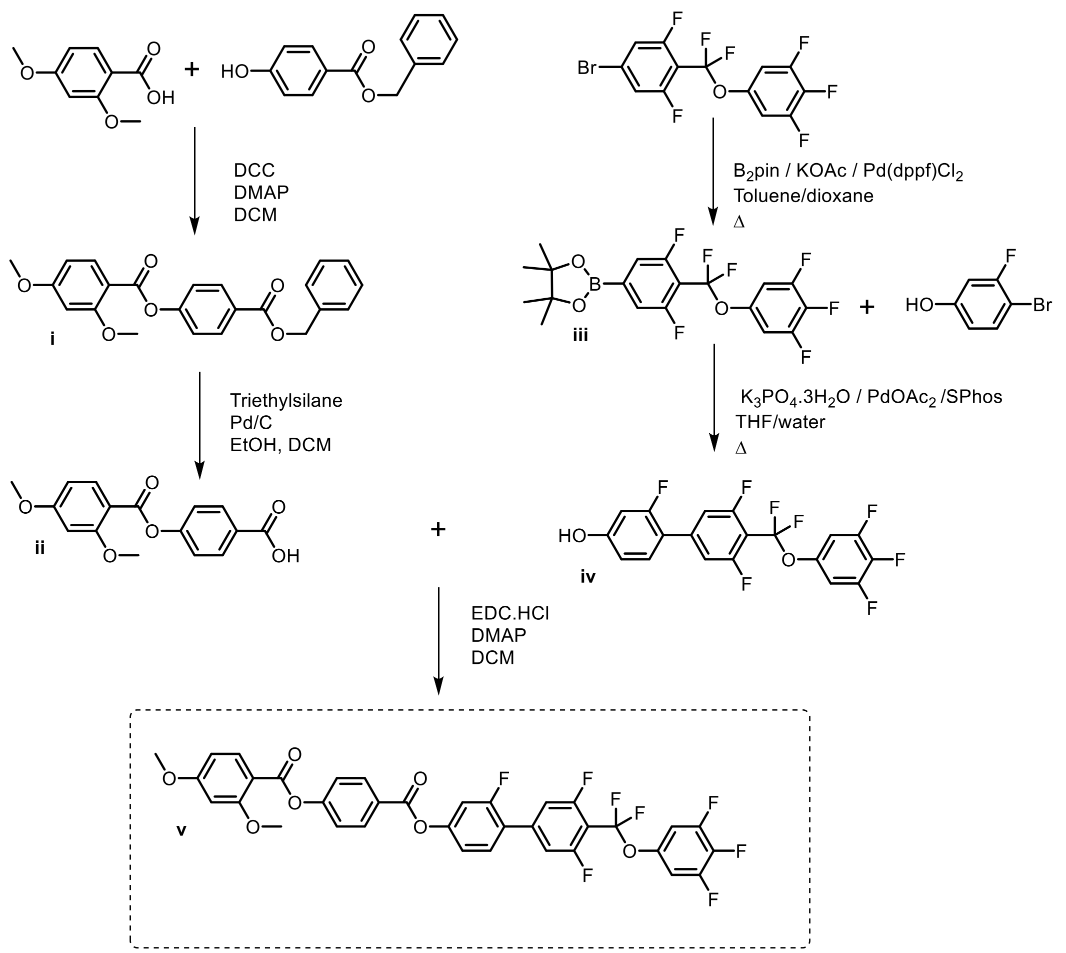

Scheme 1: Synthetic route to the new material reported here.

Compounds **i,**[5] **ii,** [5] **iii,**[6] and **iv**[7] have been previously reported.

Benzyl ester i

2,4-dimethoxy benzoic acid (1.190 g, 6.6 mmol, 1.1 eq) and *N,N'*-dicyclohexylcarbodiimide (DCC) (1.730 g, 8.4 mmol, 1.4 eq) were dissolved in DCM (50 ml) and stirred for 10 minutes. Benzyl 4-hydroxybenzoate (1.371 g, 6 mmol, 1 eq) and 4-dimethylaminopyridine (DMAP) (74 mg, 0.6 mmol, 0.1 eq) were added and the reaction was left stirring at room temperature overnight. The reaction was filtered to remove the dicyclohexylurea and the solvent removed *in vacuo*. The crude product was recrystallised from ethanol to yield the product as a white solid. (1.27 g, 54 %).

^{1}H NMR (400 MHz, $CDCl_3$) δ 8.13 (d, *J* = 8.6 Hz, 2H), 8.07 (d, *J* = 8.7 Hz, 1H), 7.39 (overlapping multiplets, 5H), 7.28 (d, *J* = 8.6 Hz, 2H), 6.56 (dd, *J* = 8.7, 2.2 Hz, 1H), 6.53 (d, *J* = 2.2 Hz, 1H), 5.37 (s, 2H), 3.91 (s, 3H), 3.89 (s, 3H). ^{13}C NMR (101 MHz, $CDCl_3$) δ 165.81, 165.20, 162.95, 162.39, 154.97, 135.99, 134.58, 131.17 (2C), 128.60 (2C), 128.25, 128.17 (2C), 127.25, 122.02 (2C), 110.61, 104.86, 98.98, 66.74, 56.02, 55.60.

Acid ii

Under an argon atmosphere triethylsilane (1.6 ml, 10 mmol, 10 eq.) was added dropwise to a stirred solution of **i** (393 mg, 1 mmol) and 5 % Pd/C (80 mg) in ethanol (3 ml) and DCM (3 ml). The reaction was stirred for 5 minutes after addition was complete, then filtered through celite and the solvent removed *in vacuo*. The crude product was washed with hexane to yield the product as a white powder. (300 mg, quant.) R_f 0.22 (DCM)

^{1}H NMR (400 MHz, $CDCl_3$) δ 8.15 (d, *J* = 8.5 Hz, 2H), 8.08 (d, *J* = 8.8 Hz, 1H), 7.32 (d, *J* = 8.5 Hz, 2H), 6.57 (dd, *J* = 8.8, 2.2 Hz, 1H), 6.54 (d, *J* = 2.2 Hz, 1H), 3.93 (s, 3H), 3.90 (s, 3H). ^{13}C NMR (101 MHz, DMSO-d_6) δ 167.16, 165.35, 163.00, 162.15, 154.67, 134.44, 131.27 (2C), 128.70, 122.70 (2C), 110.32, 106.14, 99.43, 56.43, 56.16.

Boronic ester iii

A solution of 5-bromo-2-(difluoro(3,4,5-trifluorophenoxy)methyl)-1,3-difluorobenzene (4.951 g 12.7 mmol), bis(pinacolato)diboron (3.35 g 12.7 mmol), anhydrous potassium acetate (3.67 g 36.7 mmol) in toluene (100 ml) and 1,4-dioxane (100 ml) was sparged with argon for 1 hr. Then the catalyst Pd(dppf)Cl_2 (280 mg, 3 mol%) was added and the reaction was stirred at 80°C for 5 hrs. The reaction was cooled to room temperature and added to 1M HCl solution. The product was extracted with toluene and the organic layer was washed three times with water and dried over $MgSO_4$. The solvent was removed under vacuum and the crude solid thus obtained was recrystallized from ethanol. Yield 2.5 g, 45 %).

^{1}H NMR (400 MHz, $CDCl_3$) δ 7.38 (d, *J* = 10.5 Hz, 2H), 6.96 (m, 2H), 1.35 (s, 12H). ^{13}C NMR (101 MHz, $CDCl_3$) δ 160.81, 160.78, 160.78, 158.23, 158.20, 152.26, 152.26, 152.20, 152.15, 152.15, 152.10, 149.76, 149.76, 149.71, 149.65, 149.65, 149.60, 144.72, 144.61, 144.58, 144.52, 144.47, 139.79, 139.63, 139.63, 139.48, 137.29, 137.14, 136.99, 122.83, 120.18, 120.18, 118.17, 118.14, 118.12, 118.12, 117.96, 117.95, 117.92, 117.92, 111.65, 111.42, 111.19, 107.52, 107.45, 107.35, 107.28, 84.86, 24.80.

Phenol iv

Intermediate **iii**, (1.108 g, 2.5 mmol) 4-bromo-3-fluorophenol, (0.587 g, 2.7 mmol) and potassium phosphate trihydrate (2.32 g, 8.7 mmol) were dissolved in THF (20 ml) and distilled water (1 ml), sparged with argon, and refluxed for 1 hour. Palladium acetate (33 mg, 0.15 mmol) and S-Phos (101 mg, 0.25 mmol) were added, and the reaction was refluxed for 4 hours. The mixture was cooled to RT, acidified with 1 M HCl, extracted with three portions of DCM and the organic layers were combined, dried over magnesium sulfate and the solvent removed *in vacuo*. The crude product was recrystallised from hexane to yield the desired product. (404 mg, 38 %).

^{1}H NMR (400 MHz, $CDCl_3$) δ 7.31 (t, *J* = 8.6 Hz, 1H), 7.16 (d, *J* = 11.0 Hz, 2H), 6.99 (m, 2H), 6.72 (m, 2H), 5.39 (s, 1H). ^{13}C NMR (101 MHz, $CDCl_3$) δ 161.55, 161.16, 161.10, 159.05, 158.62, 158.60, 158.54, 157.86, 157.74, 152.28, 152.23, 152.17, 152.12, 149.79, 149.74, 149.68, 149.63, 141.51, 141.40, 141.30, 139.80, 139.65, 139.50, 137.31, 137.16, 137.00, 130.84, 130.79, 120.22, 118.06, 117.94, 112.76, 112.73, 112.70, 112.52, 112.49, 112.45, 112.16, 112.13, 107.55, 107.48, 107.37, 107.31, 104.21, 103.96. ^{19}F NMR (376 MHz, $CDCl_3$) δ -61.72 (t, *J* = 26.3 Hz, 2F), -111.03 (td, *J* = 26.3, 10.3 Hz, 2F), -114.51 (t, *J* = 10.3 Hz, 1F), -132.53 (dd, *J* = 20.9, 8.0 Hz, 2F), -163.22 (tt, *J* = 20.9, 5.8 Hz. 1F).

Target ester v

Intermediate acid **iii** (71 mg, 0.24 mmol) and EDC.HCl (62 mg, 0.32 mmol) were dissolved in 10 ml DCM and stirred for 5 minutes. Phenol **iv** (90 mg, 0.21 mmol) and DMAP (3 mg, 0.02 mmol) were added, and the mixture stirred overnight and monitored by TLC (R_f 0.58 DCM). The reaction mixture was washed 3 times with water and the organic layer dried over magnesium sulfate and removed *in vacuo*. The crude solid was purified by column chromatography (gradient elution, 50/50 DCM: hexane → DCM) and triturated with hexane. Yield 63 mg (42 %).

^{1}H NMR (400 MHz, $CDCl_3$) δ 8.26 (d, *J* = 8.6 Hz, 2H), 8.10 (d, *J* = 8.7 Hz, 1H), 7.50 (t, *J* = 8.5 Hz, 1H), 7.39 (d, *J* = 8.5 Hz, 2H), 7.27 – 7.14 (overlapping multiplets and chloroform, 5H), 7.04 – 6.97 (m, 2H), 6.58 (d, *J* = 9.1 Hz, 1H), 6.55 (s, 1H), 3.94 (s, 3H), 3.91 (s, 3H). ^{13}C NMR (101 MHz, $CDCl_3$) δ 165.35, 163.97, 162.80, 162.50, 161.22, 161.16, 160.78, 158.65, 158.59, 158.27, 155.83, 152.39, 152.30, 152.28, 152.25, 152.20, 152.15, 149.81, 149.76, 149.70, 149.65, 140.90, 140.87, 140.77, 140.77, 140.67, 140.64, 139.84, 139.71, 139.69, 139.54, 137.35, 137.20, 137.05, 134.63, 131.82, 130.56, 130.52, 125.77, 123.28, 123.25, 123.13, 122.44, 120.11, 120.08, 118.50, 118.47, 113.24, 113.21, 113.18, 113.00, 112.97, 112.93, 111.07, 110.81, 110.35, 107.58, 107.52, 107.41, 107.34, 104.94, 98.98, 56.04, 55.62. ^{19}F NMR (376 MHz, $CDCl_3$) δ -61.82 (t, *J* = 26.4 Hz, 2F), -110.45 (td, *J* = 26.4, 10.4 Hz, 2F), -113.83 – -113.93 (m, 1F), -132.37 – -132.55 (m, 2F), -163.12 (tt, *J* = 20.9, 5.9 Hz, 1F).

HRMS (ESI) m/z Calculated for $C_{35}H_{20}O_7F_8$:
$[M+H]^+$ theoretical mass: 705.11540, found 705.11657, difference 1.65 ppm.
$[M+Na]^+$ theoretical mass: 727.09735, found 727.09838, difference 1.42 ppm.

IR (ν_{max}/cm^{-1}) 3101, 3074, 3005, 2926, 2847, 1741, 1710, 1611, 1519, 1233, 1135, 1037.

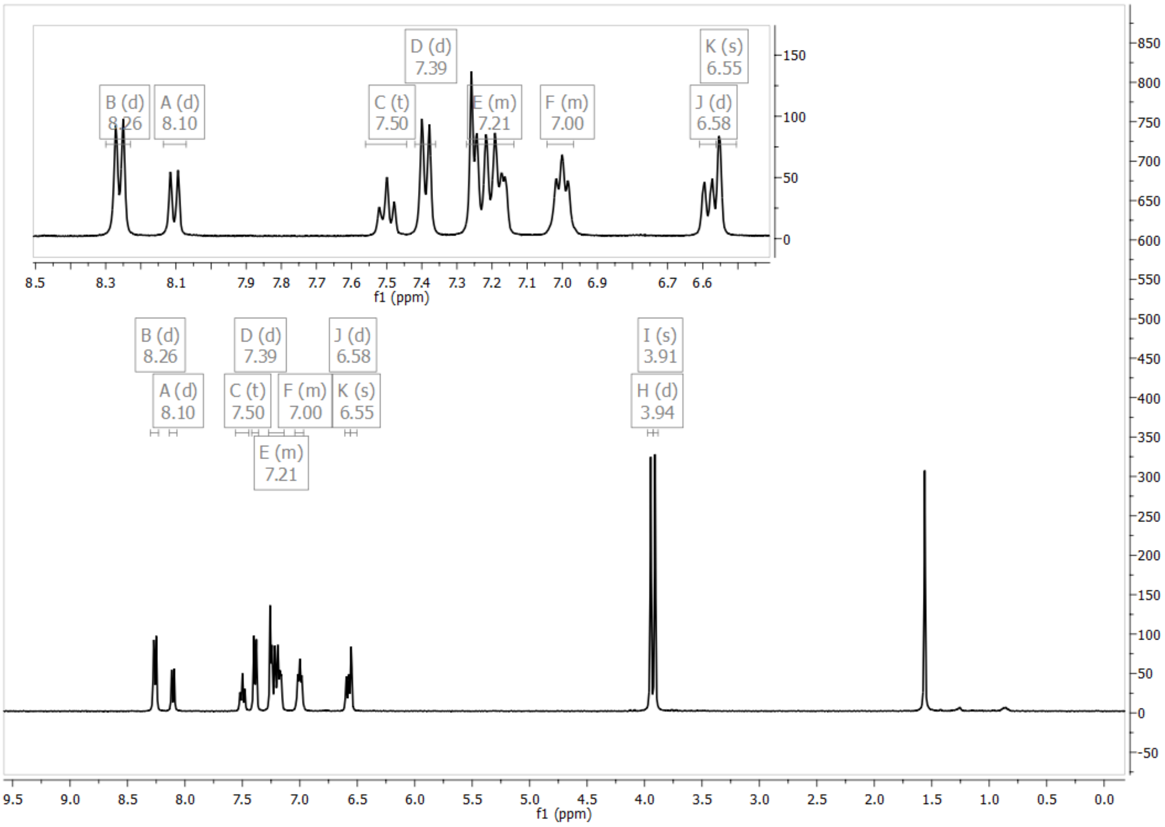

*Figure S1: ^{1}H NMR spectrum of compound **v** in $CDCl_3$.*

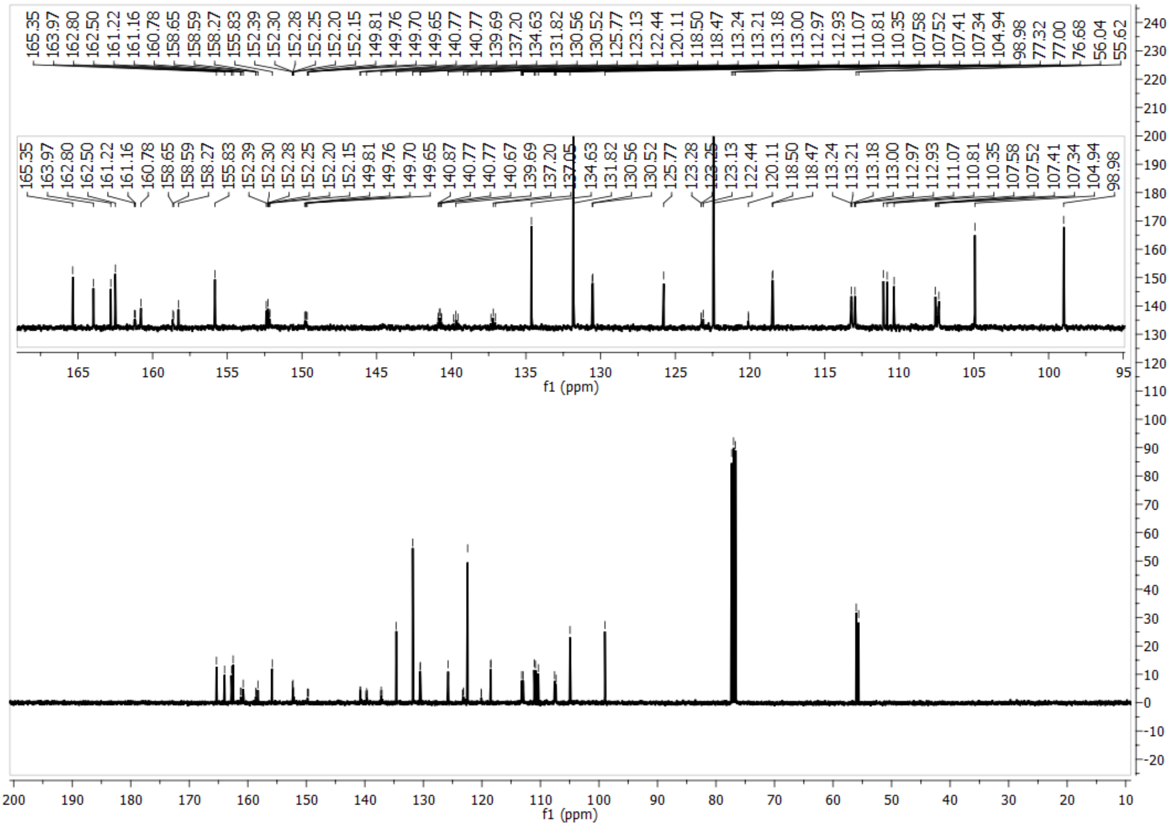

*Figure S2 ^{13}C NMR spectrum of compound **v** in $CDCl_3$.*

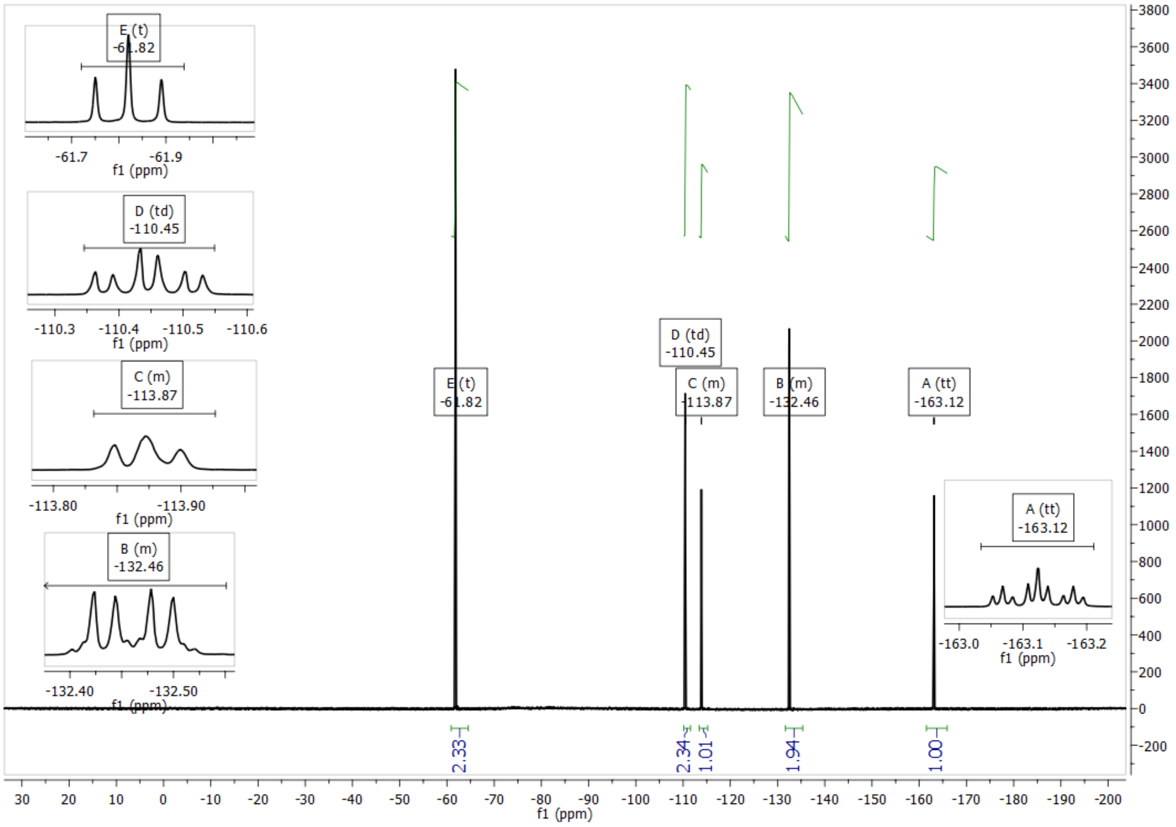

Figure S3: ^{19}F NMR spectrum of compound ***v*** *in $CDCl_3$.*

Single crystal X-ray diffraction

The material crystallized in centrosymmetric P-1 space group in triclinic system, with one independent molecule in the asymmetric unit. There were no traces of the solvent molecules in voids in this crystal structure. There is a static disorder concerning the position of the fluorine F8 with 93% of the major component with F8 bound to C19 and 7% of the minor component (F8a bound to C15, Figure S4).

The molecule in the crystal structure is fully stretched with no co-planar phenyl rings. The terminal rings #1 and #5 are nearly perpendicular to the closest-connected rings #2 and #4 accordingly, while the three middle rings are rotated at about 40° with respect to each other (Fig. S4 and Table S1), in order to avoid short F … F and F … H intramolecular contacts. The ester groups are also not co-planar with the attached phenyl rings: O2 and O4 atoms are out of plane of the ring #4 and #5 accordingly by 24.83(17)° and 23.45(17).

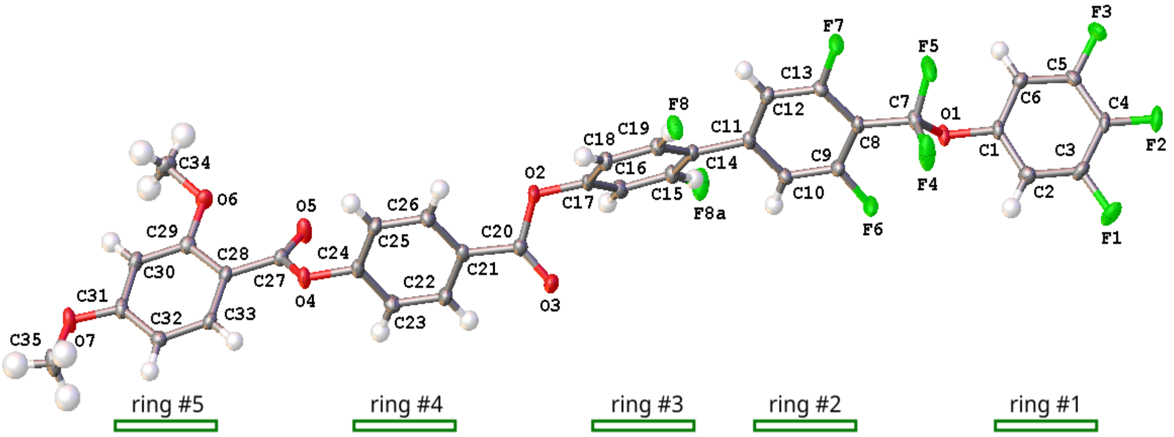

Figure S4. Determined structure of molecule with the applied numbering scheme. H atom numbers are the same as those of the closest covalently bound C atom. Atomic displacement parameters represented at 50% probability level. The minor variant of disorder labelled as F8a. Aromatic rings in the structure have been assigned numbers based on the order of C atoms.

Table S1. Angles between the planes of the phenyl rings.

Phenyl rings	Angle [°]
#1 - #2	126.81(6)
#2 - #3	39.91(6)
#3 - #4	36.53(6)
#4 - #5	107.36(6)

The most important intermolecular interactions have been identified using interaction energy estimation with UNI potential[8,9] within Mercury[10] and illustrated in Figure S5. Notably, the two strongest interactions involve intermolecular π … π stacking of the ring #5 with its symmetry-related equivalent and #2 with #4, while the third occurs between the molecules related by translation and appears to arise from maximizing the number of C – H … F interactions (Tables S2, S3).

As a consequence, the molecules all align approximately with the crystallographic [1-1-1] direction but do not form separate columns or layers in the crystal structure (Figure S6).

Table S2. Geometry of intermolecular π ... π interactions in the crystal structure.

phenyl rings involved	inter-planar angle [°]	inter-centroid distance [Å]	lateral shift [Å]
#1---#1(-1+X,1+Y,+Z)	3.422(6)	3.877(6)	1.773(6)
#2---#4(-X,1-Y,1-Z)	0.000	3.745(7)	1.786(7)
#5---#5(4-X,-2-Y,-Z)	0.000	3.514(6)	1.102(7)

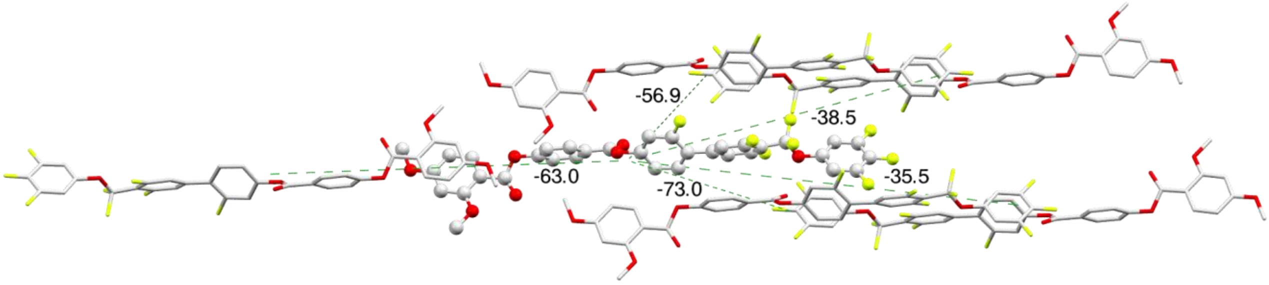

Figure S5. The most important intermolecular interactions in the crystal structure represented as dashed lines between the centers of interacting molecules. Among the three strongest interactions, two rely on π ... π stacking.

(a)

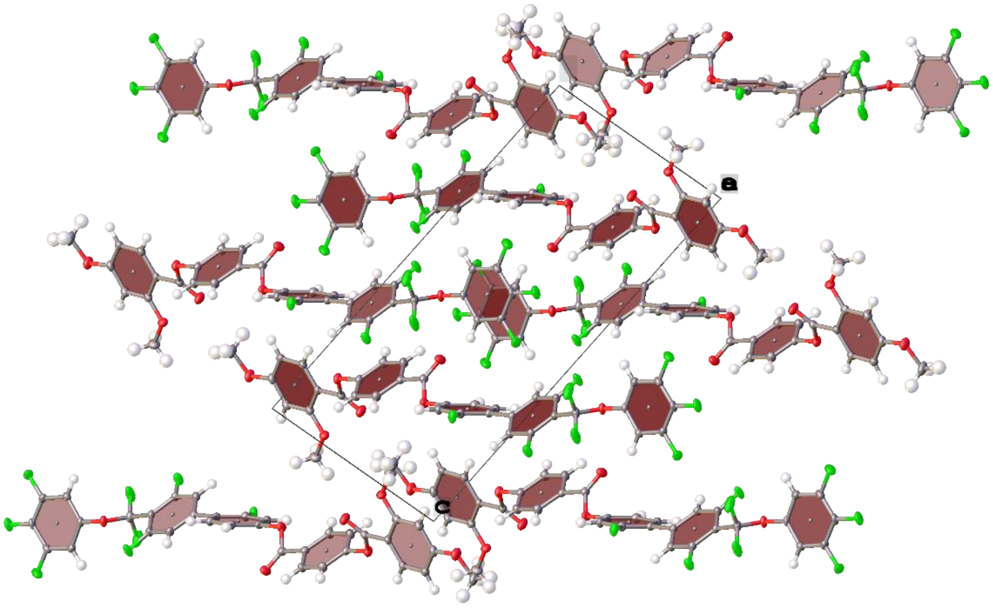

(b)

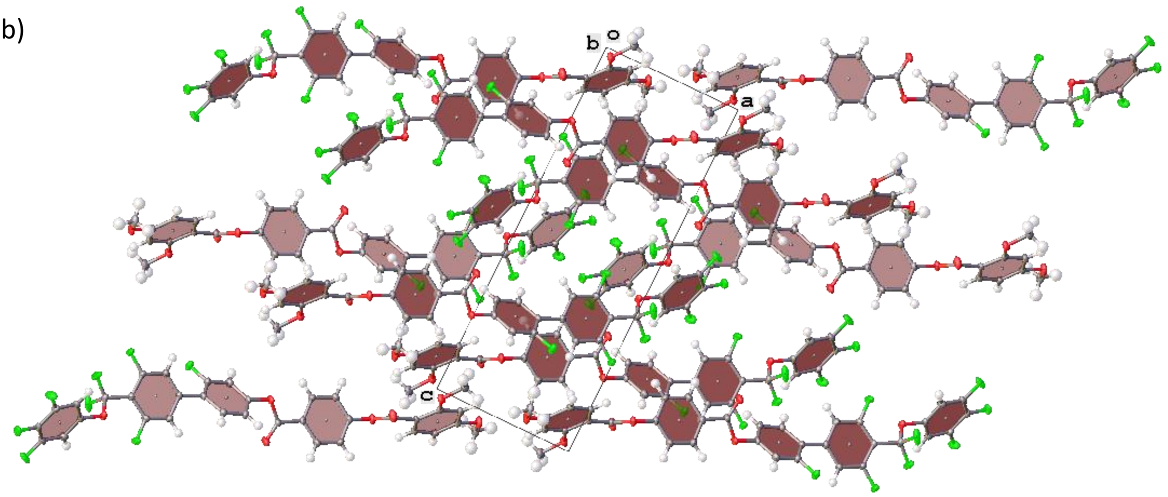

(c)

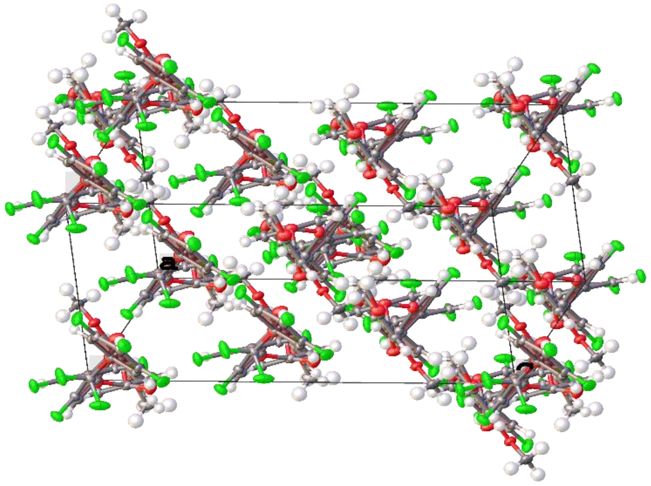

Figure S6. Visualization of crystal packing in selected directions: (a) view along [100], (b) view along [010] and (c) view along [1-1-1]. Phenyl rings outlined in brown.

Table S3. The strongest intermolecular interactions.

	Intermolecular Distance [Å]	Energy [kJ/mol]	
1	11.16	-72.9	π … π interactions (ring #5)
2	20.83	-63.0	π … π interactions (rings #2 and #4)
3	7.82	-56.8	C – H … F and C – H … π
4	19.78	-38.5	C – H … F and C – H … π
5	24.16	-35.5	π … π interactions (ring #1)

The final crystal structure was deposited with the CCDC (**CCDC Number 2375621**).

Detailed information on X-ray data and crystal structure model are summarized in Table S4.

Table S4. Crystal data and structure refinement for the studied compound

Empirical formula	$C_{35}H_{20}F_8O_7$
CCDC code	2375621
Formula weight	704.532
Temperature/K	120.00(10)
Crystal system	triclinic
Space group	P-1
a/Å	7.8233(3)
b/Å	9.3769(4)
c/Å	20.2777(8)
α/°	83.455(3)
β/°	89.797(3)
γ/°	80.365(3)
Volume/Å^3	1456.82(10)
Z	2
ρcalcg/cm^3	1.606
μ/mm^{-1}	0.145
F(000)	716.7
Crystal size/mm^3	0.211 × 0.098 × 0.037
Radiation	Mo Kα (λ = 0.71073)
2Θ range for data collection/°	4.04 to 58.9
Index ranges	$-10 \le h \le 10$, $-12 \le k \le 12$, $-26 \le l \le 27$
Reflections collected	21214

Independent reflections	7454 [R_{int} = 0.0426, R_{sigma} = 0.0477]
Data/restraints/parameters	7454/1/463
Goodness-of-fit on F^2	1.056
Final R indexes [I>=2σ (I)]	R_1 = 0.0517, wR_2 = 0.1078
Final R indexes [all data]	R_1 = 0.0700, wR_2 = 0.1165
Largest diff. peak/hole / e Å^{-3}	0.46/-0.34

Intermolecular energy estimation was based on an empiric potential,[8,9] Potential = A*exp(-Br) – Cr(-6) with unified parameters according to Table S5.

Table S5. Unified (UNI) pair-potential parameters:

atom1	atom2	A	B	C
F7	F7	170916.4	4.22	564.8
F7	O4	182706.1	3.98	868.3
F7	O5	182706.1	3.98	868.3
F7	C31	196600.9	3.84	1168.8
F7	H10	64257.8	4.11	248.4
O4	O4	195309.1	3.74	1335.0
O4	O5	195309.1	3.74	1335.0
O4	C31	393086.8	3.74	2682.0
O4	H10	295432.3	4.82	439.3
O5	O5	195309.1	3.74	1335.0
O5	C31	393086.8	3.74	2682.0
O5	H10	295432.3	4.82	439.3
C31	C31	226145.2	3.47	2418.0
C31	H10	120792.1	4.10	472.8
H10	H10	24158.0	4.01	109.2

Supplementary figures

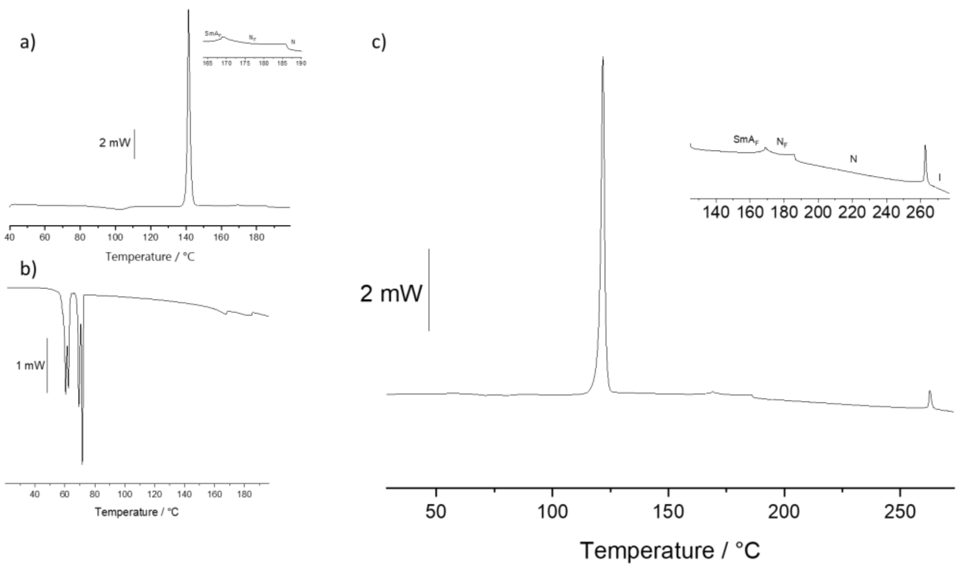

***Figure S7:** DSC traces for the new material studied: a) first heating cycle, to avoid decomposition the temperature range was limited to 190°C b) cooling cycle, c) second heating cycle.*

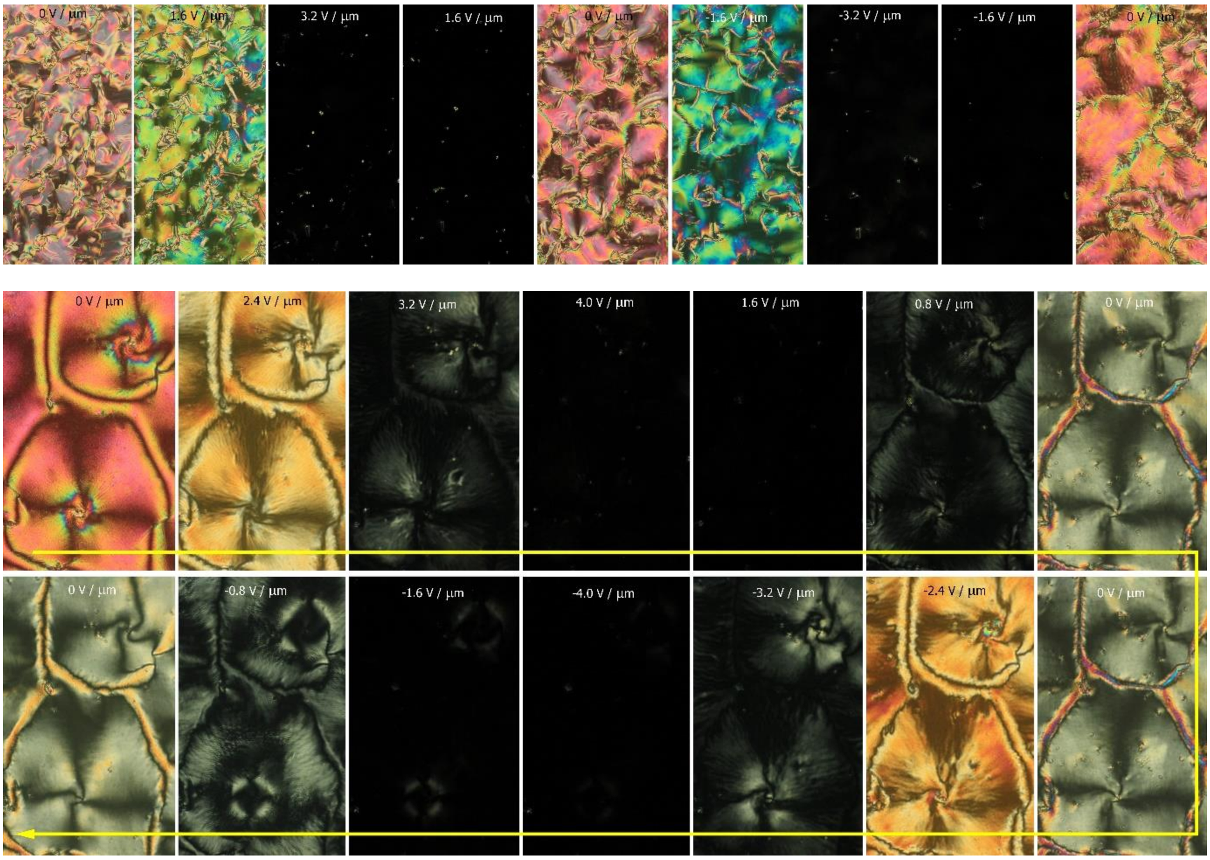

***Figure S8**: Optical texture changes under applied dc electric field in SmA_F (upper row) and SmC_F (bottom rows) phases. In both phases, above a certain threshold voltage a homeotropic state is obtained, with polarization oriented along the applied field.*

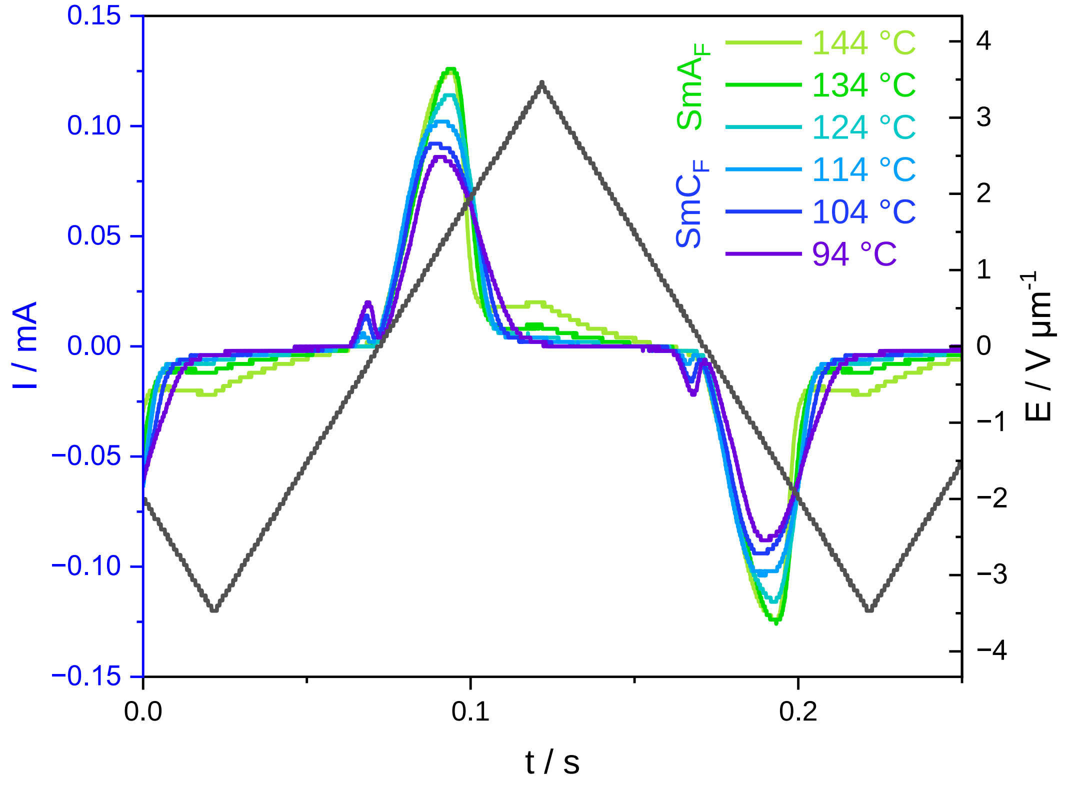

***Figure S9**: The switching current recorded in the SmC_F and SmA_F phases (blue and green lines) under application of triangular wave voltage (black line). On heating, the small peak observed at low voltages in the SmC_F phase decreases and disappears on entering the SmA_F phase. The main peak narrows and grows in height on heating through both smectic phases.*

Supplemental References

[1] Rigaku-Oxford-Diffraction, Crysalispro software system. Version 1.171.38.46, Rigaku Oxford Diffraction, **2019**.

[2] O. V. Dolomanov, L. J. Bourhis, R. J. Gildea, J. a. K. Howard, H. Puschmann, *J. Appl. Crystallogr.* **2009**, *42*, 339–341.

[3] L. J. Bourhis, O. V. Dolomanov, R. J. Gildea, J. A. K. Howard, H. Puschmann, *Acta Crystallogr. Sect. Found. Adv.* **2015**, *71*, 59–75.

[4] M. J. Frisch, G. W. Trucks, H. B. Schlegel, G. E. Scuseria, M. A. Robb, J. R. Cheeseman, G. Scalmani, V. Barone, G. A. Petersson, H. Nakatsuji, X. Li, M. Caricato, A. V. Marenich, J. Bloino, B. G. Janesko, R. Gomperts, B. Mennucci, H. P. Hratchian, J. V. Ortiz, A. F. Izmaylov, J. L. Sonnenberg, Williams, F. Ding, F. Lipparini, F. Egidi, J. Goings, B. Peng, A. Petrone, T. Henderson, D. Ranasinghe, V. G. Zakrzewski, J. Gao, N. Rega, G. Zheng, W. Liang, M. Hada, M. Ehara, K. Toyota, R. Fukuda, J. Hasegawa, M. Ishida, T. Nakajima, Y. Honda, O. Kitao, H. Nakai, T. Vreven, K. Throssell, J. A. Montgomery Jr., J. E. Peralta, F. Ogliaro, M. J. Bearpark, J. J. Heyd, E. N. Brothers, K. N. Kudin, V. N. Staroverov, T. A. Keith, R. Kobayashi, J. Normand, K. Raghavachari, A.

P. Rendell, J. C. Burant, S. S. Iyengar, J. Tomasi, M. Cossi, J. M. Millam, M. Klene, C. Adamo, R. Cammi, J. W. Ochterski, R. L. Martin, K. Morokuma, O. Farkas, J. B. Foresman, D. J. Fox, **2016**.
[5] R. J. Mandle, *Chem. - Eur. J.* **2017**, *23*, 8771–8779.
[6] J. Karcz, N. Rychłowicz, M. Czarnecka, A. Kocot, J. Herman, P. Kula, *Chem. Commun.* **2023**, *59*, 14807–14810.
[7] J. Karcz, J. Herman, N. Rychłowicz, P. Kula, E. Górecka, J. Szydlowska, P. W. Majewski, D. Pociecha, *Science* **2024**, *384*, 1096–1099.
[8] A. Gavezzotti, *Acc. Chem. Res.* **1994**, *27*, 309–314.
[9] A. Gavezzotti, G. Filippini, *J. Phys. Chem.* **1994**, *98*, 4831–4837.
[10] C. F. Macrae, I. J. Bruno, J. A. Chisholm, P. R. Edgington, P. McCabe, E. Pidcock, L. Rodriguez-Monge, R. Taylor, J. van de Streek, P. A. Wood, *J. Appl. Crystallogr.* **2008**, *41*, 466–470.
